# Sprouty4 is epigenetically upregulated in human colorectal cancer

**DOI:** 10.1080/15592294.2022.2145068

**Published:** 2022-11-16

**Authors:** Alexei J. Stuckel, Shuai Zeng, Zhen Lyu, Wei Zhang, Xu Zhang, Urszula Dougherty, Reba Mustafi, Tripti Khare, Qiong Zhang, Trupti Joshi, Marc Bissonnette, Sharad Khare

**Affiliations:** a Department of Medicine, Division of Gastroenterology and Hepatology, University of Missouri, Columbia, Missouri, 65212, USA; b Bond Life Sciences Center, University of Missouri, Columbia, Missouri, 65201, USA; c Department of Electrical Engineering and Computer Science, University of Missouri, Columbia, Missouri, 65201, USA; d Department of Preventive Medicine and the Robert H. Lurie Comprehensive Cancer Center, Northwestern University Feinberg School of Medicine, Chicago, Illinois, 60611, USA; e Department of Medicine, University of Illinois, Chicago, Illinois, 60607, USA; f Department of Medicine, Section of Gastroenterology, Hepatology and Nutrition; the University of Chicago, Chicago, Illinois, 60637, USA; g Institute for Data Science and Informatics, University of Missouri, Columbia, Missouri, 65211, USA; h Department of Health Management and Informatics; School of Medicine, University of Missouri, Columbia, Missouri, 65212, USA; i Harry S. Truman Memorial Veterans’ Hospital, Columbia, Missouri, 65201, USA

**Keywords:** Colorectal cancer, Sprouty4, gene expression, DNA methylation, DNA hydroxymethylation

## Abstract

Sprouty4 (SPRY4) has been frequently reported as a tumor suppressor and is therefore downregulated in various cancers. For the first time, we report that SPRY4 is epigenetically upregulated in colorectal cancer (CRC). In this study, we explored DNA methylation and hydroxymethylation levels of *SPRY4* in CRC cells and patient samples and correlated these findings with mRNA and protein expression levels. Three loci within the promoter region of *SPRY4* were evaluated for 5mC levels in CRC using the combined bisulfite restriction analysis. In addition, hydroxymethylation levels within *SPRY4* were measured in CRC patients. Lastly, DNA methylation and mRNA expression data were extracted from CRC patients in multiple high-throughput data repositories like Gene Expression Omnibus and The Cancer Genome Atlas. Combined *in vitro* and *in silico* analysis of promoter methylation levels of *SPRY4* clearly demonstrates that the distal promoter region undergoes hypomethylation in CRC patients and is associated with increased expression. Moreover, a decrease in gene body hydroxymethylation and an increase in gene body methylation within the coding region of *SPRY4* were found in CRC patients and correlated with increased expression. SPRY4 is epigenetically upregulated in CRC by promoter hypomethylation and hypermethylation within the gene body that warrants future investigation of atypical roles of this established tumor suppressor.

## Key policy highlights

- SPRY4 gene expression is increased in colorectal cancer
- The *SPRY4* promoter loses DNA methylation, and the gene body gains DNA methylation in colorectal cancer
- The gene body of *SPRY4* loses DNA hydroxymethylation

## Introduction

The Sprouty (SPRY) gene family encodes receptor tyrosine kinase inhibitors that play an essential role in many biological processes, including embryogenesis and stem cell maintenance. Knock down (KD) studies of Sprouty4 (SPRY4) in human embryonic stem cells resulted in promoting cellular differentiation comprising all three germ layers [1]. In a Van der Woude syndrome mice model, a germline mutation in *SPRY4* contributed to dysregulation of periderm differentiation and function, leading to abnormal oral epithelial adhesions [2]. Moreover, pertaining to regulating progenitor cell growth, *SPRY4* was identified as an evolutionary conserved target of the WNT/β-catenin signalling pathway, specifically via T cell factor/lymphoid enhancer factor (TCF/LEF) transcription factor (TF) binding within the promoter region [3].

Notably regarded as a tumor suppressor in many cancers, SPRY4 is downregulated in glioblastoma, endometrial adenocarcinoma, perihilar cholangiocarcinoma (PHCC), breast carcinoma, melanoma, and also prostate, ovarian, and lung cancers [4–11]. In addition, clinical data have correlated low expression of SPRY4 with a poor prognosis in PHCC, adult myeloid leukaemia, and colorectal cancer (CRC) [6,12,13]. In contrast, SPRY4 is upregulated and may serve an oncogenic function in gastrointestinal stromal tumors (GIST) [14,15]. Furthermore, SPRY4 proteins, compacted into nano-sized vesicles, target and reprogram neighbouring cells when released as circulating exosomes from GIST-derived tumor cells [15].

In cancer and other human diseases, SPRY4 is dysregulated by several various biological mechanisms. This includes deviant expression of upstream effector molecules that regulate SPRY4 signalling. For example, in melanoma cells, upstream matrix metallopeptidase 2 (MMP2)/RAC1 signalling is increased, which results in negatively regulating the tumor suppressor function of SPRY4 [8]. Moreover, the tumor-suppressing function of SPRY4 that is triggered by upstream peroxisome proliferator-activated receptor gamma signalling is lost in non-small-cell lung cancer [16]. In terms of SPRY4 mutants, only two disease-associated variants of SPRY4 have been characterized. In hormonal disorders like Kallmann syndrome, an amino acid change occurs at position 241, where serine is converted into tyrosine, resulting in SPRY4's inability to negatively regulate epithelial growth factor signalling while simultaneously increasing its negative regulator function on fibroblast growth factor (FGF) signalling [17]. In addition, SPRY4 variant c.701C>T,p.Thr234Met results in increased expression, leading to increased cell viability and colony formation in familial nonmedullary thyroid cancer cells [18].

Post-transcriptional degradation of SPRY4 transcripts has also been documented as a mechanism contributing to the tumor phenotype observed in non-small-cell lung cancer [19]. Furthermore, several TFs have been reported to directly bind to the promoter region and regulate SPRY4 in a variety of cancer cells. TFs that regulate SPRY4 include nuclear respiratory factor-1 [20], specific protein 1 [21], and ETS proto-oncogene 1 (ETS1) [22]. SPRY4 is also epigenetically dysregulated in cancer cells by several biological mechanisms. For instance, miR-411-5p, miR-1908, and miR-181 microRNAs downregulate SPRY4 by directly targeting and degrading SPRY4 transcripts in an assortment of cancer cells [11,23–25]. Likewise, SPRY4 suppression occurs in hepatocellular carcinoma drug-resistant patients, where histone deacetylase 4 (HDAC4) modifies the histone configuration within the *SPRY4* promoter region, resulting in a heterochromatin (silenced) state [26]. In addition, the promoter of *SPRY4* remains susceptible to aberrant DNA methylation in some cancer types. For example, in prostate cancer patients, the promoter of *SPRY4* is hypermethylated, leading to downregulated expression [9]. In addition, promoter hypermethylation of *SPRY4* was observed in a large cohort study of patients with familial testicular cancer [27]. In summary, the studies showcase the various biological mechanisms by which SPRY4 is dysregulated in cancer.

We hypothesize that changes in DNA modifications of methylation of 5-methyl cytosine (5mC) and 5-hydroxymethylcytosine (5hmC) alter SPRY4 expression in CRC patients. In this study, for the first time, we report the overexpression of SPRY4 in several large CRC patient cohort studies and correlate our findings with alterations of 5mC and 5hmC in *SPRY4*.

## Materials and methods

### Human tissue collection and isolation of colonocytes from colonic mucosa

Adenocarcinomas and adjacent normal-appearing colonic mucosa samples were provided by the Department of Surgical Pathology at The University of Chicago [IRB protocol (10-209-A)]. Colonocytes were isolated as previously described [28–31]. Patient demographics include CRC individuals between 36 and 91 y of age with an assortment of confounding labels including smoker, drinker, diabetic, and tumor grade status. Patient clinical information can be found in **Supplementary Table I**.

### Cell culture

CRC cell lines were obtained from the American Type Culture Collection (ATCC) and grown in a medium supplemented with 10% foetal calf serum I (Thermo Fisher Scientific, Waltham, MA, USA). SW480 cells were grown in Leibovitz’s L-15 medium (ATCC, Manassas, VA, USA). HCT116 cells were grown in high-glucose DMEM medium (Thermo Fisher Scientific, Waltham, MA, USA). RKO and Caco2 cells were grown in ATCC-formulated Eagle’s Minimum Essential Medium (ATCC, Manassas, VA, USA).

### Combined bisulfite restriction analysis

Genomic DNA (gDNA) was extracted and eluted in water from human tissue samples and CRC cell lines (HCT116, RKO, Caco2, SW480) using the DNeasy Blood and Tissue Kit (Qiagen, Germantown, MD, USA). To obtain bisulfite-converted DNA, gDNA (~200 ng) was treated with sodium bisulfite using the EZ DNA Methylation-Gold Kit (Zymo, Irvine, CA, USA) per the manufacturer’s instructions. Bisulfite-converted DNA (~200 ng) was then PCR amplified under standard thermal conditions (15 min hot start 95°C (94°C denaturation for 30s, 56°C annealing temperature, and 72°C extension for 45 cycles) followed by 72°C final extension for 10 min) in a 25 μL reaction using the PyroMark PCR kit (Qiagen, Germantown, MD, USA). The bisulfite-specific primers with sequences designed by MethPrimer software are shown in **Supplementary Figure I** [32].

*Region #1: Distal promoter region [2CpGs] BstUI*

Forward primer: (5’->3’)GGGTGGATTATAAGGTTAGGAGTTTA

Reverse primer: (5’->3’)AAATAACACAATCAAAACCATTTT

*Region #2: Distal promoter region [1CpG]Taq1-v2*

Forward primer: (5’->3’)GGTTGGGATTTTAGAGGTAGGTAGT

Reverse primer: (5’->3’)AAACAACTATACCCATTTTTCTAAC

*Region #3: Proximal promoter region [6CpGs]BstUI*

Forward primer: (5’->3’)TGTTGTAATTATTGTTTGGGAAAAT

Reverse primer: (5’->3’)AATAAAACTTAAACCAATCCCAACA

PCR products were digested in a 25 μL reaction by either BstUI (60°C) or Taq1-v2 (37°C) restriction enzyme for 4 h. Restriction digests were then resolved on a 2.5% agarose gel with ethidium bromide – BstUI: cuts 5'CGCG’3 sequence and Taq1-v2: cuts 5'TCGA’3 sequence [33]. Combined bisulfite restriction analysis (COBRA) gels serve as qualitative data in so by comparing the band intensity differences between control and tumor samples. For instance, an increase in band intensity relating to the fragmented bands below the PCR product for one sample compared to a sample with less intense fragmented bands indicates an increase in methylation in the first hypothetical sample. This analysis hinges on the fact that the aforementioned restriction enzymes target and indirectly digest only methylated PCR products into visible fragments/bands observed on agarose gel.

### Western blotting

Proteins in the sample lysates from different colon cancer cell lines were separated by sodium dodecyl sulfate–polyacrylamide gel electrophoresis on 10% Criterion-XT BioRad gels, and the separated proteins were transferred to BioRad nitrocellulose membrane. Membranes were then blocked with 5% bovine serum albumin (BSA) at room temperature for 1 h followed by overnight incubation with primary SPRY4 rabbit polyclonal antibody at 1:500 dilution (Abcam Cat#ab59785) and β-actin mouse monoclonal antibody at 1:1000 (Cell Signalling Cat# 3700S) at 4°C. Next day, membranes were incubated for 1 h at room temperature with appropriate horseradish peroxidase-coupled secondary antibodies. Signals were detected by using the Super signal® West femto maximum sensitivity substrate (Pierce), and images were visualized by BioRad ChemiDoc XRS imager.

### Bioinformatics and statistical analyses

#### Gene expression and methylation analyses

High-throughput data from CRC patients were extracted from The Cancer Genome atlas (TCGA) and Gene Expression Omnibus (GEO) datasets (GSE166427 and GSE24514) [34]. A Student’s *t*-test was performed on normalized methylation β-values from 450K methylation microarray data derived from control and tumor CRC patient samples archived in TCGA. GEO2R tool from the National Center for Biotechnology Information (NCBI) was utilized to determine SPRY4 expression differences in both GEO datasets using the Benjamini & Hochberg (FDR) method with a *p*-value <0.05. Seaborn software was used to generate boxplots displaying DNA methylation and mRNA expression distributions [35]. 450K DNA methylation probe and histone/ZFX ChIP-seq data from Caco2 and HCT116 cells were extracted from the Encyclopaedia of DNA Elements (ENCODE) [36]. TCGA patient clinical information can be found in **Supplementary Table II**. Gene-level copy number variation was downloaded and parsed by R package TCGAbiolinks [37]. GISTIC2 package in R was utilized to determine gene-level copy number variations in CRC patients archived in TCGA [38]. R software (package ggplot2) was used to generate the boxplot showing gene expression in different gene-level copy number groups.

#### Hydroxymethylation data analysis

As for 5hmC-modified locations in the gene body, 12 pairs of 5hmC-Seal data were downloaded from tumor and adjacent tissue samples from patients with CRC (GSE89570). Patient libraries were assembled through an enrichment process of 5hmC DNA fragments [39]. The normalized counts from DESeq2 by library size were log2 transformed and corrected for batch effect using linear regression. A paired *t*-test was conducted to evaluate whether the 5hmC modification levels in *SPRY4* promoter and gene body were different between tumors and adjacent tissues (*p*-value <0.05).

## Results

### SPRY4 is upregulated in colon adenocarcinomas

**Figure 1.**
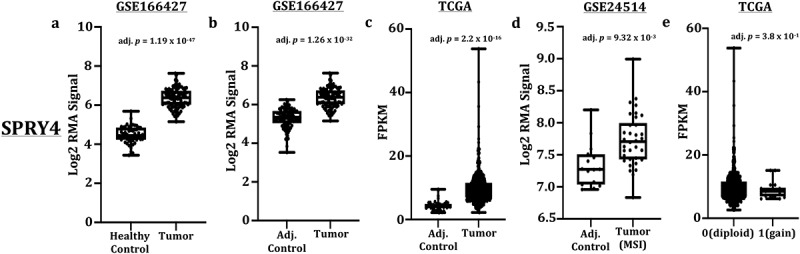
*Transcript expression of SPRY4 in colorectal cancer (CRC) patients*. (a) Increased SPRY4 transcripts in adenocarcinomas compared to healthy mucosa from Gene Expression Omnibus (GEO) dataset (GSE166427). Increased SPRY4 transcripts in adenocarcinomas compared to adjacent control from (b) GEO dataset (GSE166427) and (c) The Cancer Genome Atlas (TCGA). (d) Increased SPRY4 transcripts in microsatellite instable (MSI) CRC patients compared to adjacent control. (e) No statistical difference in transcript expression (FPKM) was observed between *SPRY4*-diploid CRC patients (0) and CRC patients with copy number gains of *SPRY4* (1).

In order to accurately assess SPRY4 expression in CRC, patient’s whole-genome RNA-derived microarray datasets were extracted from large CRC cohort studies registered in the NCBI’s GEO data repository. In addition, RNA-seq data from CRC patients catalogued in TCGA were parsed and analysed for normalized read count differences between CRC patients and control samples. An increase in SPRY4 transcripts was identified in patients with colon adenocarcinomas compared to both adjacent colon and healthy mucosa controls (GSE166427) (Figure 1a,b). CRC patients derived from TCGA also exhibited increased SPRY4 mRNA expression compared to adjacent control samples (Figure 1c). Furthermore, CRC tumors classified as microsatellite instable also displayed increased SPRY4 transcript expression in a separate study (GSE24514) (Figure 1d).

Increased copy number of *SPRY4* may contribute to the increased expression observed in this study, and was therefore investigated in CRC patient adenocarcinomas classified as *SPRY4*-diploid compared against patients with copy number gains of *SPRY4*. Utilizing CRC patients in TCGA data repository, we found no group differences in SPRY4 transcript expression between patient adenocarcinomas identified as *SPRY4*-diploid or patients with copy number gains of *SPRY4* (Figure 1e). Overall, two separate CRC gene expression studies and CRC patients analysed in TCGA revealed increased SPRY4 mRNA expression in colon adenocarcinomas.

### DNA methylation regulates SPRY4 expression in colon adenocarcinomas in the distal promoter region

**Figure 2.**
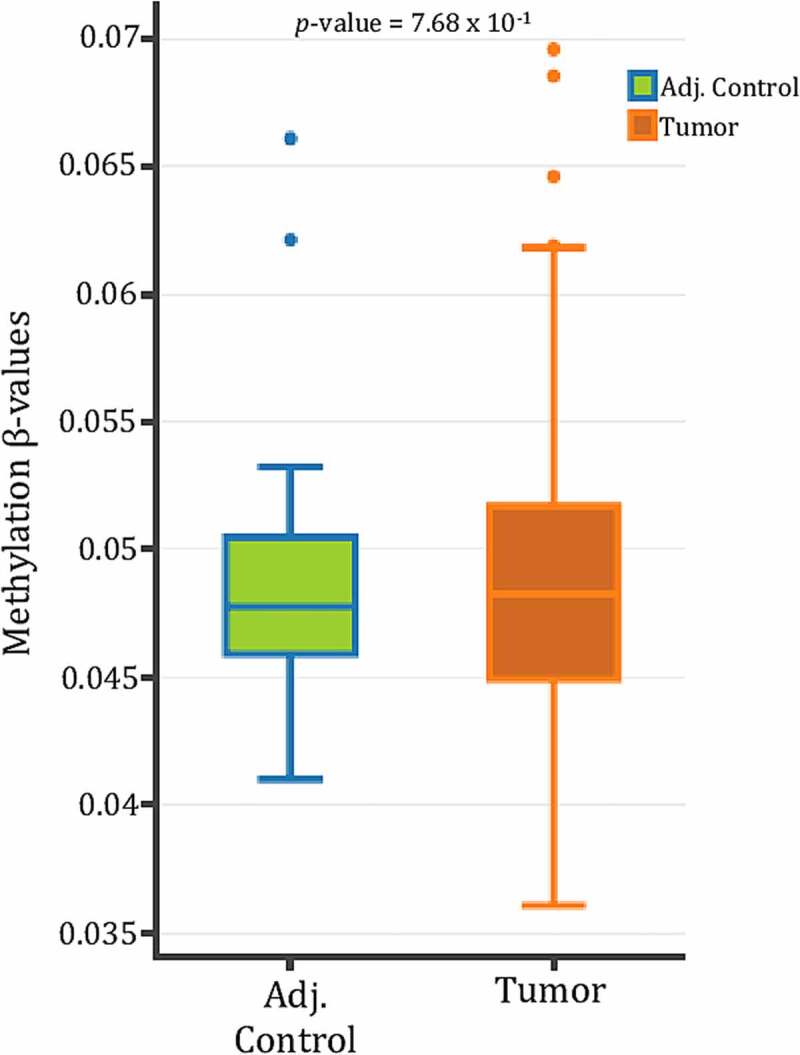
*SPRY4 proximal promoter methylation in colorectal cancer (CRC) patients. SPRY4* promoter methylation β-value comparison of eight probes spanning the 5'UTR/1^st^ Exon to a small CpG island (~1500 bps from TSS) between CRC patients archived in The Cancer Genome Atlas (TCGA) and their corresponding adjacent control samples. No statistical significance between groups was observed. Note: DNMIVD is a database in which boxplot data results from methylation queries on the SPRY2 gene promoter were automatically generated.

**Figure 3.**
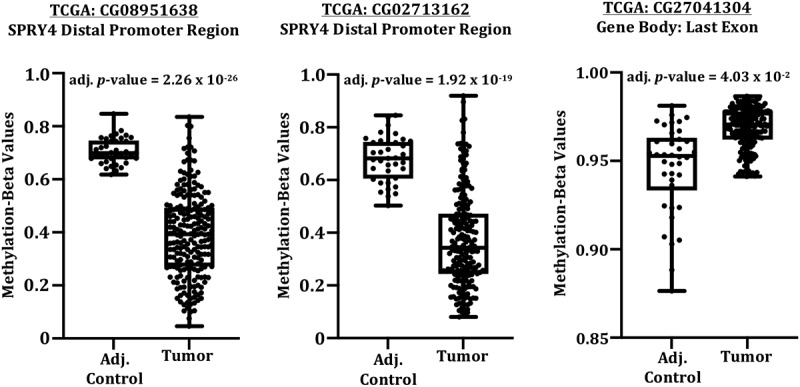
*DNA methylation 450K probe data in SPRY4 in colorectal cancer patients archived in The Cancer Genome Atlas*. Three statistically differentially methylated CpG probes between adjacent control colon and colon adenocarcinomas located in the [distal promoter regions: CG08951638 & CG02713162] and the [gene body: CG27041304] of *SPRY4* (shown left to right). *Note*: A decrease in mean methylation β-values in both distal promoter regions of *SPRY4* and an increase in methylation β-values in the gene body of *SPRY4* in colon adenocarcinomas.

Investigative reports in regard to unveiling mechanisms regulating SPRY4 in CRC are severely lacking. We therefore queried previously compiled *SPRY4* 5mC 450K CG probe data within the proximal 5’ promoter region from CRC patients in TCGA utilizing the DNA methylation interactive visualization database (DNMIVD) [40]. No differences in β-methylation values between colon adenocarcinomas and adjacent controls existed within the proximal region of SPRY4's promoter, which spans two CpG islands near the transcriptional start site (TSS) (Figure 2). Furthermore, the displayed methylation β-values within the proximal promoter region are too low to even be considered partially methylated in both groups (methylation β-value <2.0). However, in the remaining probes spanning *SPRY4*, we found impressively disparate losses of 5mC of two CG probes located in the distal promoter regions (1500 bps and 1000 bps upstream of TSS) and hypermethylation of one CG probe within the last exon of *SPRY4* (gene body) (Figure 3). In short, differential methylation and detectable levels of 5mC within the proximal promoter region were not evident; rather, hypomethylation within the distal promoter regions and gene body hypermethylation may be associated with increased SPRY4 gene expression changes in CRC.

Given that our *in silico* analysis demonstrated that *SPRY4* promoter hypomethylation inversely correlated with increased mRNA expression in large CRC datasets prompted us to confirm differential methylation within the distal promoter regions of *SPRY4* in CRC patient samples. We selected two distal promoter regions #1 and #2 that each contains CGs within close proximity (~100 bps) of the two previously analysed hypomethylated CG probes found in CRC patients from TCGA. The positions of the two distal regions were also chosen in part because they overlap putative TF-binding sites belonging to known transcriptional activator Zinc Finger Protein X-Linked (ZFX) nested within enriched euchromatin (active) histone marker H3K27ac, as revealed by ChIP-seq data from the Encyclopaedia of DNA Elements (ENCODE) repository in CRC cell line HCT116 [36]. Furthermore, tumor grades and differentiation status greatly vary in patients (*n* = 13) where COBRA was performed (Supplementary Table I). The results indicate that SPRY4 methylation status was independent of tumor grades and differentiation status in a small subset analysed. Results also corroborate with TCGA analyses where patients had different tumor grades and differentiation status (Supplementary Table II).

**Figure 4.**
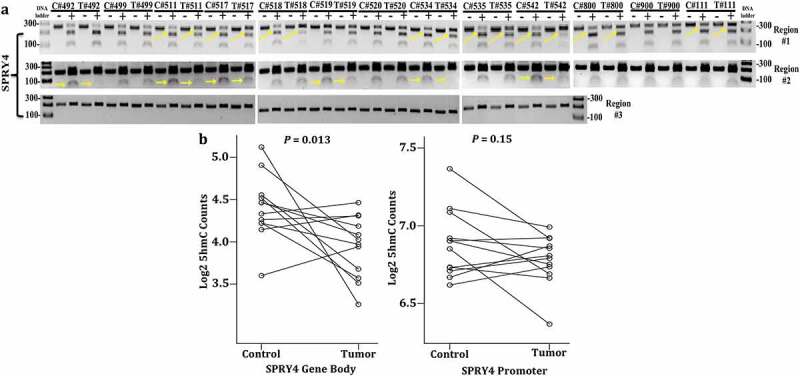
*Determining DNA methylation and hydroxymethylation levels of SPRY4 in colorectal cancer (CRC) patients*. (a) Combined bisulfite restriction assay (COBRA) results of three regions of interest located in the promoter region of *SPRY4* from 13 CRC patients (T# patient ID number) and adjacent normal colonocytes (C# patient ID number). Each sample was run on two lanes: lane (1) bisulfite-treated PCR-amplified DNA without restriction enzyme digestion, which served as a reference control for unmethylated CpG and lane, and (2) bisulfite-treated PCR-amplified DNA digested with a restriction enzyme recognizing amplicons containing a 5'mCpG sequence. + Lane: PCR product treated with restriction enzyme lane, – Lane: PCR product from untreated sample. Yellow arrows serve to highlight differential methylation between matched control and tumor samples for a given patient. For instance, in region #1, the PCR band product (top band) in experimental lane 40 (#542 patient tumor) was less enzymatically digested and therefore higher in intensity (less methylated) than the top PCR band in lane 38 (#542 patient control). Again, in region #2 of patient #542, enzymatic digestion of PCR products was less evident in the tumor sample vs. the control sample, indicating hypomethylation (loss of methylation) in regions #1 and #2 in CRC patient #542. (b) 5-hydroxymethylcytosine (5hmC) abundance in *SPRY4* gene bodies in colon cancers and matched adjacent mucosa (*n* = 12 samples, *p* < 0.05, paired Student’s *t*-test): No change in mean log2 fold change values of 5hmC in the *SPRY4* gene promoter and a statistically significant decrease in gene body 5hmC in colon tumors (Tumor) compared to normal colon (Control).

DNA was then extracted from 13 colon adenocarcinomas and adjacent normal-appearing colonocytes and subjected to methylation analysis by COBRA. In distal promoter region #1, 8 out of 13 CRC patients exhibited hypomethylation (losses of 5mC) compared to adjacent control samples (Figure 4a). Similarly, in distal promoter region #2, 6 out of 13 CRC patients displayed losses of 5mC. Collectively, 10 out of 13 CRC patients demonstrated distal promoter hypomethylation within regions #1 and/or #2 (Figure 4a). To corroborate our TCGA findings of no differential methylation within the proximal promoter region of *SPRY4*, COBRA was conducted in region #3 located within a CpG island 500 bps upstream of the TSS. No evidence of 5mC was detected in a total of six CpGs, and therefore, no differential methylation between adenocarcinomas and control samples was found in region #3 (Figure 4a). In general, our COBRA data confirm predominate losses of 5mC in the distal promoter regions of *SPRY4*, which correlates with overall increased transcript expression observed in the CRC population.

### Decreased 5hmC marks occur within the gene body of SPRY4 in CRC

Commonly, changes in 5hmC deposition within the promoter and gene body regions inversely correlate with changes in 5mC [39]. Hence, the existing 5hmC next-generation sequencing profiles from colon adenocarcinomas and matched control tissues were parsed to examine changes in 5hmC levels within the promoter and gene body of *SPRY4*. No statistical change in 5hmC deposition was observed in the promoter region of *SPRY4*. However, a decrease of 5hmC within the gene body of *SPRY4* was detected in colon adenocarcinomas (Figure 4b). This observed loss of gene body 5hmC inversely correlates with our findings of increased 5mC of ‘CG27041304’ probe within the gene body of *SPRY4* in TCGA CRC patients (Figure 3).

### Distal promoter regions of SPRY4 are hypermethylated in CRC cells

**Figure 5.**
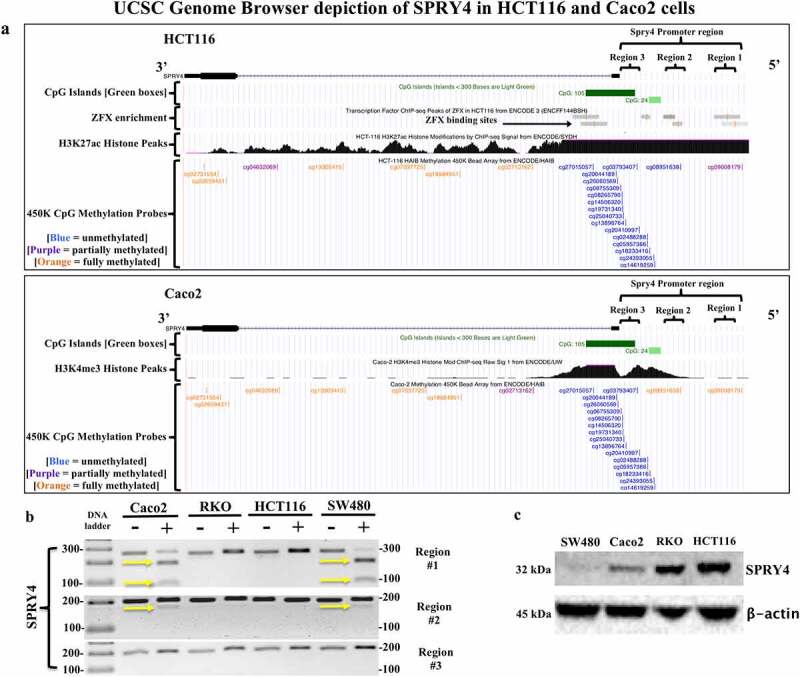
*Evaluating SPRY4 promoter methylation and protein expression in colorectal cancer (CRC) cells*. (a) UCSC Genome Browser (hg19 build) depiction of *SPRY4* in two CRC cell lines: (top) HCT116 and (bottom) Caco2. *SPRY4* contains two CpG islands (green), and according to ChIP-seq data, in HCT116 cells, there are ZFX transcription factor (TF)-binding sites located in region #1 and #2 (grey bars = TF-binding enrichment). Note the extensive methylation that is present in regions #1 and #2 of *SPRY4* in Caco2 cells as indicated by two orange (full methylation) vertical tick marks. In contrast, HCT116 cells contain probable partial methylation spanning the distal end of region #1 but no methylation in region #2, as indicated by a blue (no-methylation) vertical tick mark. Lastly, ChIP-seq peaks associated with active transcriptional regulatory regions of *SPRY4* are represented by histone markers (H3K27ac) and (H3K4me3) in CRC cell lines HCT116 and Caco2, respectively. (b) COBRA results of three regions of interest located in *SPRY4* from: four CRC cell lines: Caco2, RKO, HCT116, and SW480 cells. The yellow arrows indicate the presence of 5mC: region #1 shows partial PCR product enzymatic digestion in Caco2 and SW480 cells and region #2 displays slight PCR product enzymatic digestion, indicating partial methylation of *SPRY4* in Caco2 and SW480 cells in both regions. Region #3 shows no evidence of methylation in all CRC cell lines. (c) Western blot analysis of SPRY4 protein expression in four CRC cell lines that inversely correlate with 5mC status in regions #1 and #2.

The UCSC genome browser depiction of the epigenetic landscape of *SPRY4* in CRC cell lines HCT116 and Caco2 determined that distal promoter regions #1 and #2 were potential regions of interest, considering differential methylation between Caco2 and HCT116 cells exists in both regions as indicated by 450K methylation probe data from ENCODE (Figure 5a). For example, *SPRY4* regions #1 and #2 in Caco2 cells are considered fully methylated (orange CpG probe) compared to partially methylated (purple CpG probe) and unmethylated (blue CpG probe) regions #1 and #2 in HCT116 cells. Thus, COBRA was utilized to explore the same two distal promoter regions in CRC cell lines. We found that *SPRY4* regions #1 and #2 were methylated in Caco2 and SW480 cells as indicated by enzymatic digestion of PCR products by CpG-specific restriction enzymes (Figure 5b). For Caco2, our COBRA data corroborate with ENCODE’s findings of 5mC within both regions #1 and #2. In contrast, HCT116 cells did not display 5mC within regions #1 and #2. However, ENCODE data suggest that region #1 may contain partial 5mC in the distal sequence of region #1 in HCT116 cells. We also tested the proximal promoter region of *SPRY4* (region #3) and found no evidence of 5mC in any CRC cell line tested in this study. This finding is corroborated by ENCODE’s methylation data, which shows no evidence of 5mC within the proximal promoter region spanning both CpG islands of *SPRY4* in HCT116 and Caco2 cells. We then found that *SPRY4* promoter hypermethylation in distal promoter regions #1 and #2 in Caco2 and SW480 cells inversely correlated with decreased protein expression in comparison to *SPRY4* in unmethylated and highly expressed RKO and HCT116 cells (Figure 5c). Therefore, 5mC status within the distal promoter regions likely influences SPRY4 gene expression as demonstrated in CRC cells.

## Discussion

Currently, only a tumor suppressor function for SPRY4 has been posited in CRC, where SPRY4 mRNA underexpression was reported in a limited number of CRC patients [13]. However, we are the first to report increased SPRY4 transcripts as analysed in TCGA and two independent GEO datasets containing large CRC sample sizes. This inversely correlated with our findings of hypomethylation in the distal promoter regions (regions #1 and #2) of *SPRY4*, whereas no evidence of 5mC was detected in the proximal promoter region (region #3). These findings contradict Zhou et al.’s finding of reported *SPRY4* promoter hypermethylation in a limited number of CRC patients [13], where genomic coordinates or approximations of where DNA methylation was tested in the *SPRY4* promoter region were not provided. In addition, bisulfite-specific primers were also not cited, and therefore, we were unable to replicate their reported results.

The lack of 5mC detection within the proximal promoter region #3 in our CRC patient samples by COBRA was corroborated by TCGA’s findings of undetectable β-methylation values in both CRC and control patient samples. Compellingly, ENCODE’s 450K-methylation array data for CRC cell lines HCT116 and Caco2 and our COBRA data for HCT116, Caco2, RKO, and SW480 clearly demonstrate that the proximal promoter region of *SPRY4* is also unmethylated in CRC cells and therefore rule out this locus as potential contributor in regulating SPRY4 gene expression. Rather, our COBRA studies indicated that distal promoter regions #1 #2 are hypermethylated in Caco2 and SW480 cells and correlate with decreased SPRY4 protein expression. Convincingly, our findings of *SPRY4* distal promoter hypermethylation and decreased protein expression align with Guo et al.’s finding of overall decreased SPRY4 expression in SW480 cells [41]. In fact, in SW480 cells, SPRY4 contains marginally increased 5mC levels in region #1 and is observantly less expressed than in Caco2 cells.

This disparity in methylation and gene expression levels between primary cancer cells and cell lines seems counterintuitive. Our past studies have made examples out of this phenomenon for genes CXCR4 and SPRY2 in CRC [42,43]. Both of these genes greatly differ in methylation and expression between various CRC cell lines, whereas promoter hypomethylation and increased gene expression was consistently observed in patient tumor samples. For example, among the Sprouty genes aberrantly expressed in CRC, we have previously shown that SPRY2 is also methylated and downregulated in several CRC cell lines [43]. However, in human CRC samples, we have shown that both the Sprouty genes (SPRY2 and SPRY4) are hypomethylated in the distal promoter regions and upregulated. Thus, to our advantage using deductive reasoning based on combined SPRY4 patient and cell line data, we can shift our focus away from unmethylated promoter region #3 and conduct future studies on distal promoter regions #1 and #2 that likely epigenetically regulate SPRY4 in both patient samples and cell lines by means of both hypo- and hypermethylation, respectively.

Interestingly, losses of 5mC within the distal regions of *SPRY4* occur within putative ZFX-binding sites. ZFX is increased in CRC patients and often associated with poorer prognosis [44]. In addition, ZFX preferably binds promoters of oncogenes containing CpG islands like *SPRY4* and is considered a transcriptional activator in many cancer cells [45]. Therefore, ZFX may be a potential therapeutic target for future studies in relation to SPRY4 gene regulation in CRC. Also, in this context, we previously identified CCCTC-binding factor (CTCF)-binding enrichment within the distal promoter region of SPRY2 that was hypomethylated in CRC patients and may therefore serve as a transcriptional activator. SPRY2 was initially thought of as a tumor suppressor. However, we conversely found that SPRY2 was indeed an oncogene in CRC. Recent reports have cited increased expression of SPRY2 involved in chronic colitis and inflammatory bowel disease [46]. Given the homology and similarities in the epigenetic architecture and gene expression pattern for SPRY2 and SPRY4 in CRC, we postulate that SPRY4 may also act as an oncogene as previously demonstrated for SPRY2.

In summary, our results are the first to indicate that the distal regions of *SPRY4* undergo hypomethylation in CRC patients. Collectively, our *in silico* (*n* = 2) and COBRA (*n* = 3) analyses identified five hypomethylated CpGs in CRC patients associated with increased SPRY4 mRNA expression. Furthermore, losses of gene body 5hmC often inversely correlate with increased gene body 5mC and overall increased gene expression [47,48]. Indeed, we reported decreased gene body 5hmC and increased 5mC within the coding region of *SPRY4* in CRC. Altogether, in contrast to other cancers, SPRY4 is epigenetically upregulated and may therefore serve a different function in CRC, which is yet to be discovered. In this regard, future evaluation of adenoma tissues is necessitated as most of the key molecular alterations that lead to tumorigenesis occur early in the adenoma-to-cancer sequence. To determine whether tumor size and location have any correlation with SPRY4 methylation, a much larger COBRA cohort and sample size in TCGA, which include a vast array of differentiated tumors and sizes/stages, are needed. Furthermore, in future studies, CpG island methylator phenotype status of patients should be investigated in a larger cohort to explain whether changes in methylation status are specific to SPRY4 gene or secondary to more global alterations. Nonetheless, this study warrants further investigation of oncogenic potential and functionality of SPRY4 for targeted therapeutic interventions in CRC patients.

## Supplementary Material

Supplemental Material

## Data Availability

Patient data were extracted from The Cancer Genome Atlas data repository: COAD (Colon adenocarcinomas) and GEO (Gene Expression Omnibus). https://www.cancer.gov/about-nci/organization/ccg/research/structural-genomics/tcga&https://www.ncbi.nlm.nih.gov/geo/
