# Supplementary material for: Sprouty4 is epigenetically upregulated in human colorectal cancer
Source: Epigenetics. 2022 Nov 16;18(1):2145068. doi: 10.1080/15592294.2022.2145068 (PMC9980603; doi:10.1080/15592294.2022.2145068)

**Primer Start Size TM GC% ‘C’s Sequence**

**Region #1: F1/R1**

**Region #1: Distal promoter: putative ZFX binding site hg19[Chr5: 141,707,000-141,708,500]**


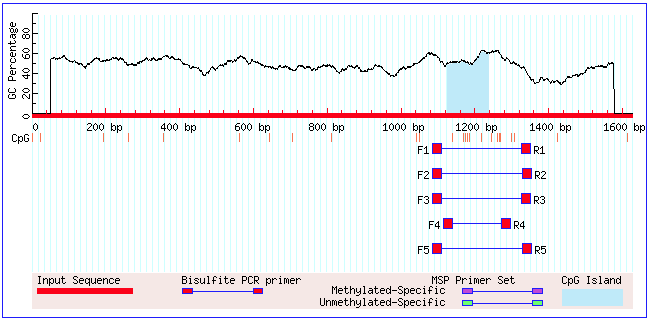

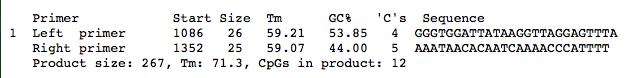


**Supplementary Figure 1. MethPrimer: Bisulfite Primer Design For *SPRY4***

**Primer Start Size TM GC% ‘C’s Sequence**

**Region #2: F5/R5**

**Region #2: Distal promoter: putative ZFX binding site hg19[Chr5: 141,705,750-141,707,000]**


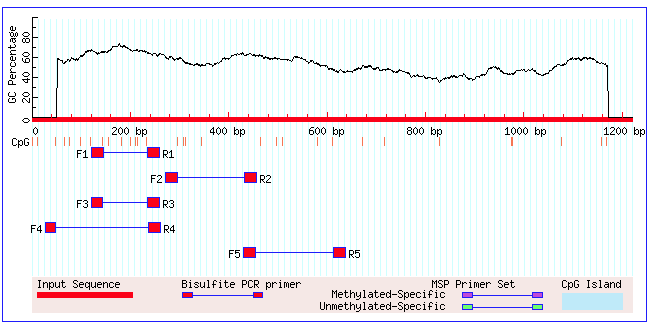

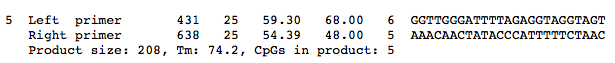


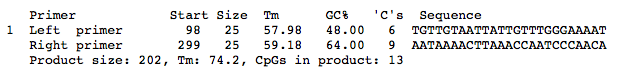


**Primer Start Size TM GC% ‘C’s Sequence**

**Region #3: F1/R1**

**Region #3: Proximal promoter CpG island: hg19[Chr5: 141,704,500-141,705,000]**


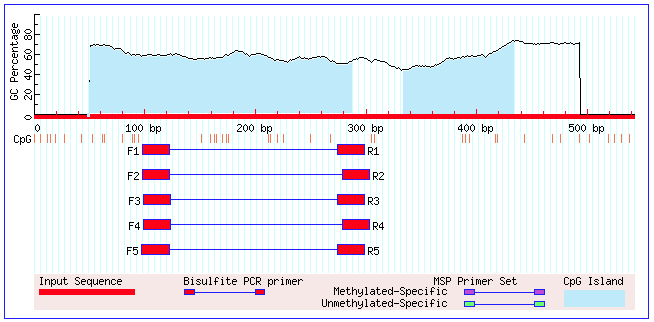

Supplement: Supplemental Material [file KEPI_A_2145068_SM1740.zip › supplement/Suppl Fig1.docx]
